# Input Layer Regularization and Automated Regularization Hyperparameter Tuning for Myelin Water Estimation Using Deep Learning

**DOI:** 10.1002/nbm.70276

**Published:** 2026-04-22

**Authors:** Mirage Modi, Shashank Sule, Jonathan Palumbo, Michael Rozowski, Griffin S. Hampton, Mustapha Bouhrara, Wojciech Czaja, Richard G. Spencer

**Affiliations:** ^1^ National Institute on Aging National Institutes of Health Baltimore Maryland USA; ^2^ Department of Mathematics University of Maryland College Park Maryland USA

**Keywords:** additive models, bilevel optimization, inverse problems, multiexponential analysis

## Abstract

We present a deep learning framework that combines classical regularization and data preprocessing to improve estimation of the myelin water fraction (MWF) in the brain from magnetic resonance relaxometry data. The proposed method is developed within the context of biexponential signal modeling, a standard approach for quantifying MWF. Building on prior work on input layer regularization (ILR), we introduce several key extensions. First, we incorporate optimal regularization hyperparameter selection using either a dedicated neural network or generalized cross‐validation (GCV), applied on a signal‐by‐signal (or pixel‐by‐pixel) basis to generate concatenated input features. Second, we extend the framework to directly estimate MWF in addition to exponential time constants. On synthetic data, the proposed architecture outperforms both conventional regularized fitting methods and standard multilayer perceptrons. When applied to in vivo brain data, it again yields superior accuracy, with GCV‐based parameter selection performing slightly better than the neural network alternative. These findings demonstrate that ILR enhances MWF estimation within the biexponential model and that classical regularization techniques, when integrated with deep learning, can substantially improve quantitative estimation of myelin content.

## Graphical abstract

We propose a novel deep learning algorithm for predicting the myelin water fraction from multiple gradient‐echo or spin‐echo pulse sequences arising in magnetic resonance relaxometry (MRR) measurements of the human brain. Our method incorporates both regularized nonlinear least squares and pure deep learning through a concatenation paradigm known as input layer regularization and improves estimation performance over both pure deep learning or pure classical estimation methods. A somewhat surprising conclusion from our experiments on clinical data shows that the best estimation method tends to “hybridize” classical and deep learning strategies and elevates the role of the generalized cross‐validation estimator as a favorable tool for combination with deep learning.

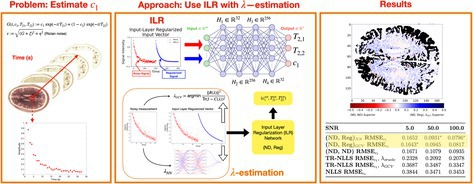

## 1 Introduction

Myelin is a lipid‐rich substance that surrounds and insulates nerve axons in the central and peripheral nervous systems, facilitating the rapid and high‐fidelity transmission of electrical signals. Therefore, quantification and mapping of myelin provides valuable insights into brain white matter. In addition, myelination in the central nervous system (CNS) is increasingly recognized as being of central importance in a spectrum of neurodegenerative disorders, including Alzheimer's disease and cognitive impairment [1], making it an important biomarker for neurodegenerative disorders such as Alzheimer's disease, multiple sclerosis (MS), and traumatic brain injury (TBI) [2, 3, 4, 5, 6]. Consequently, the problem of quantification and mapping of the myelin water fraction (MWF) using MRI remains a very active area of investigation, even after its introduction through the pioneering work of MacKay et al. more than 20 years ago [4].

There are a number of current methods for MWF mapping; relaxation‐based methods rely upon use of an MRI signal model that incorporates T2 values of both myelin‐associated water and less‐restricted water. In this paper we address multiple gradient‐echo or spin‐echo pulse sequences [7, 8, 9] where the signal observed from each voxel at an echo time t follows the following exponential model:

(1) $$s(t)=\int_{\;\mathbb{R}}e^{-t/T_{2}}p(T_{2})\;dT_{2}.$$

Here, $p(T_{2})$ represents the underlying probability density of the $T_{2}$ times and is the object of interest. In the case of MWF mapping, for the remainder of this paper, we model $p(T_{2})$ as a sum of point masses $p(T_{2})=c_{1}\delta_{T_{2,1}}+(1-c_{1})\delta_{T_{2,2}}$, representing the rapid transverse relaxation of the less‐mobile water entrapped within myelin lamellae represented by $1/T_{2,1}$ as compared with the slower rate of transverse relaxation of more‐mobile water represented by $1/T_{2,2}$. This leads to the following biexponential model for s(t) given by

(2) $$s(t;T_{2,1},T_{2,2},c_{1},c_{2})=c_{1}e^{\frac{-t}{T_{2,1}}}+(1-c_{1})e^{\frac{-t}{T_{2,2}}}.$$

In practice, the signal is observed at finitely many echo times under Rician noise (described further in Section 2). In effect, the problem is that of separating the two exponential decay signals that comprise the net acquired noise corrupted measurement s. Depending primarily upon the ratio in the decay constants of the two underlying signals, this can be an extremely challenging parameter estimation (PE) problem [10, 11]. Consequently, it has attracted several estimation techniques ranging from classical estimation theory to more modern deep learning approaches which attempt to resolve the underlying ill‐posedness of separating the two decay constants.

In this paper, we propose a novel deep learning method for biexponential signal estimation by combining deep learning with classical estimation, by improving a previously proposed deep learning technique termed input layer regularization (ILR) [12]. The idea behind ILR relies on the somewhat simple but straightforward strategy of *regularized curve fitting* where given a noisy signal, the parameter p may be estimated by fitting a biexponential curve to the data with the complexity of the parameters being penalized by a regularization term. When this regularization term is the squared norm of the parameters, this is the well‐known Tikhonov regularization (TR), and the quality of the parameters so obtained depends on the weighting between the data fidelity and regularization term, controlled by the all‐important hyperparameter λ≥0. The curve fit parameters $p_{\lambda }^{*}$ so obtained will implicitly contain information regarding p; as a consequence, a biexponential signal generated from these curve‐fitted parameters can be concatenated with the original noisy measurement to serve as an additional source of information for a neural network. It was seen empirically that this process improves the MSE for a neural network trained to estimate the time decays $T_{21},T_{22}$ given a constant MWF value.

In the original use of ILR, however, a single λ was chosen for all signals, irrespective of the underlying true parameter combination. For the three‐parameter biexponential recovery problem, a universal choice of λ tends to hinder rather than help the neural network, as different signals may require different levels of TR. Our key insight is that if this λ is chosen optimally based on the signal (with optimality being described in Section 2), then the parameters $p_{\lambda }^{*}$ obtained from the curve fit will closely approximate the underlying parameters p. The biexponential signal derived from these optimal parameters can then be considered a “denoised” or regularized version of the original signal—we concatenate this denoised or regularized version with the original noisy data and feed the concatenated pair to a neural network which outputs a parameter estimate $p_{est}$. This procedure can be viewed as augmenting the input layer of a conventional neural network with a regularized signal that contains high‐quality information about the underlying true parameters. We further expand on our method's connection to the empirical Bayes method in Section 2.6.1.

### 1.1 Contributions

Having briefly described our method, our detailed contributions are as follows:

1. We propose a novel hybrid classical and deep learning method by improving our previously published work on ILR [12] by introducing signal‐dependent selection of the regularization hyperparameter λ.
2. We explore two choices for selecting this λ optimally from the signal: (1) following [13] by using a neural network termed $\lambda_{\text{NN}}$ and (2) using generalized cross‐validation (GCV) [14], leading to the estimator $\lambda_{\text{GCV}}$. We empirically demonstrate that mean absolute error (MAE) or the $L^{1}$ loss is an effective loss function to train the network $\lambda_{\text{NN}}$ which takes a noisy signal to its optimal regularization hyperparameter termed $\lambda_{oracle}$. We show that $\lambda_{\text{NN}}$ outperforms $\lambda_{\text{GCV}}$ across all noise levels in terms of matching the underlying distribution of $\lambda_{oracle}$ (Table 2). We remark that both approaches are instances of a bilevel optimization‐based framework for studying the optimality of regularization parameters, and to the best our knowledge, the present paper is the first instance to study the optimal λ‐selection problem from this bilevel perspective for any multiexponential PE problem.
3. Next, we study the central inverse problem of estimating the MWF $c_{1}$ from the signal s. We show that on synthetically generated data our proposed method of ILR with signal‐dependent λ‐selection outperforms both classical curve fitting and purely deep learning–based approaches (Tables 3, C1, and A3).
4. We apply our techniques to in vivo brain data for two patients and three slices per patients and find consistent improvement via ILR in each slice. A significant aspect of our analysis of brain data is an initial classification of noise level at every voxel followed by voxel filtering by the Akaike information criterion (AIC) to restrict the application of our methods to voxels whose signals are better fit with a biexponential model versus a monoexponential model. Furthermore, we find that the ILR strategy with GCV‐based selection of λ outperforms ILR with NN‐based selection. This represents an application in which classical methods, including GCV, are combined with deep learning for performing challenging estimation tasks in brain data.

### 1.2 Related Work

Estimation of MWF from the transverse decay signal is known to be an ill‐posed problem and to require imposition of a prior in order to obtain meaningful results. One approach is to model the decay as the sum of two discrete decaying exponentials. Extraction of the two decay time constants can be far from trivial, but ultimately yields point estimates for the more rapidly decaying MWF and the more slowly decaying nonmyelin‐associated water. This is in keeping with other techniques yielding single, discrete values for these two water components, and in particular with the widely used mcDESPOT analysis. That is, in common with biexponential analysis as performed in this and other papers, mcDESPOT [15, 16, 17] yields a single $T_{2}$ value for myelin‐associated water and less‐restricted water, respectively, as well as single values representing corresponding component fractions.

**FIGURE 1:**
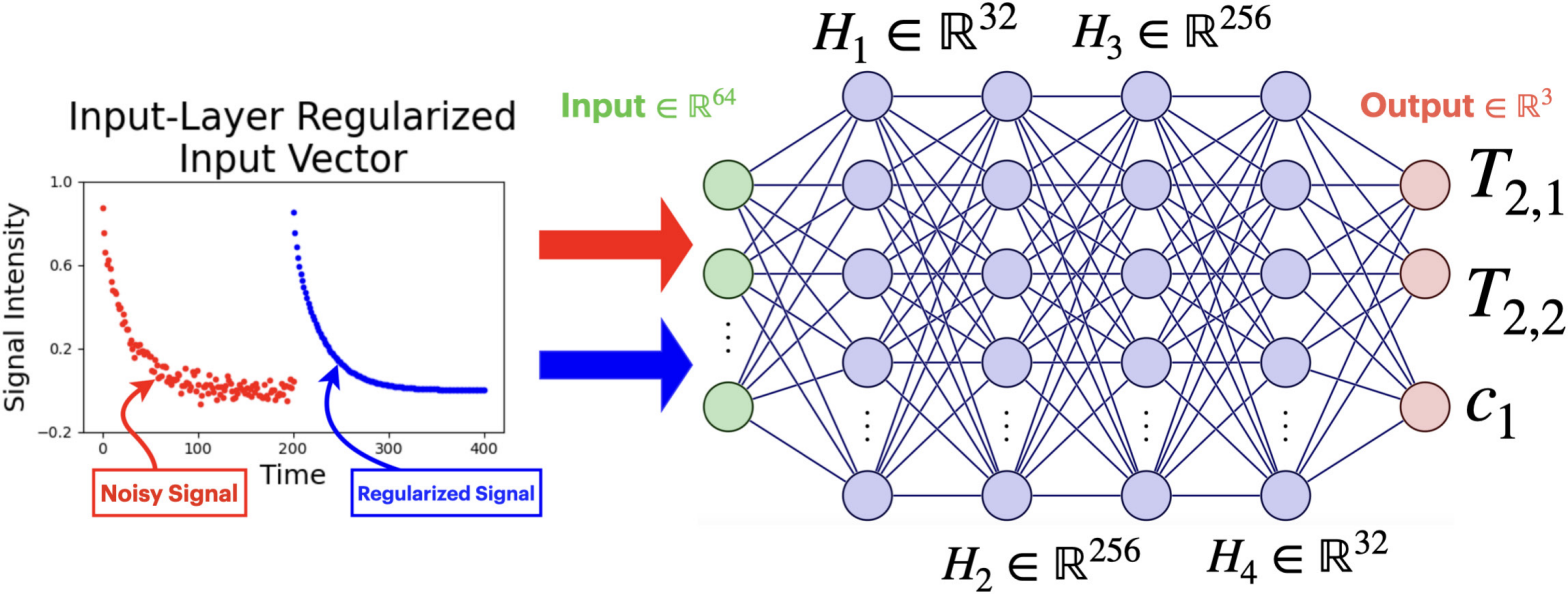
The (ND, Reg) parameter estimation network is an NN where the inputs are the concatenated vectors of noisy signal s and the signal obtained from parameters estimated by Tikhonov regularization, with regularization parameter λ(s) given by either a neural network or GCV. The parameters estimated are $c_{1},T_{2,1}$, and $T_{2,2}$.

Alternative modeling choices have also been made, in which the underlying $T_{2}$ distribution has been taken as Gaussian, Laplacian, or multi‐Gaussian, with one study showing that these model choices contribute negligibly to the estimation error in the MWF (see Figure 1 [18]). A nonparametric approach is based on the regularized nonnegative least (NNLS) method, in which there is no assumption regarding the number of exponentially decaying components [4]. Instead, the location and amplitude of the modes of the $T_{2}$ distribution, corresponding to distinct water components, are inferred from the recovered distribution. This is most commonly performed using a hard cutoff value, essentially performing a classification task in which a binary assignment to myelin‐associated water or nonmyelin‐associated water is made for each value of $T_{2}$. The cutoff serves as a hyperparameter and, depending on the morphology of the recovered distribution, can be of greater or lesser physical plausibility. As with any classification task, and perhaps especially one with a nonvalidated single value cutoff, there are associated Type I and Type II assignment errors that are typically not modeled. We have found (data not shown) that modeling the transverse decay as two discrete exponentials versus modeling that decay with the nonparametric approach yields results within a few percent of each other). In any event, the goal of this paper is not to perform a comparison of nonlinear least squares (NLLS) (biexponential and mcDESPOT) with NNLS approaches to MWF estimation but rather to describe a novel NN architecture for the biexponential model and readily generalizable to other PE problems.

There has also been steady progress in deep learning methods for denoising Rician‐noised MR images [19, 20, 21, 22, 23]. A frequent limitation with this approach is the lack of large volumes of real‐life MR images with ground truths for model training. Therefore, there also exist methods for data augmentation through synthetic generation of MR images [24]. Finally, several deep learning methods also perform the denoising and PE steps in tandem, directly mapping a noisy MR image to the underlying parameters [25, 26, 27, 28, 29, 30]. This is the approach we will use, and our approach will be benchmarked against another state‐of‐the‐art deep learning approach, model‐informed machine learning (MI‐ML) [30], in Section 4.

Thus, point‐estimator approaches to MWF quantification, as in this work, are viable and in use. In any event, our goal here is not to compare point‐estimator methods (mcDESPOT and biexponential) with approaches based on recovery of T2 distributions [31] but rather to further develop ILR for biexponential analysis.

### 1.3 Paper Organization

The outline of the paper is as follows; this also serves to collect several symbols and definitions. In Section 2, describing general features of the problem setup, we first define the biexponential model with Rician noise and outline the NLLS approach to PE in this model as an inverse problem. We note the requirement for regularization and outline methods for regularization parameter selection, including a λ selection NN, denoted $\lambda_{\text{NN}}$. Finally, we describe ILR.

In Section 3, we describe two methods of ILR implementation, based, respectively, on selection of λ via GCV or via a separate λ selection NN, denoted respectively by $\lambda_{\text{GCV}}$ and $\lambda_{\text{NN}}$. In both cases, the selection of λ is followed by the implementation of the PE NN using ILR. This involves concatenating the noisy decay (ND) vector with a version of itself regularized with either $\lambda_{\text{GCV}}$ or $\lambda_{\text{NN}}$ to form the input signal to the PE NN, with the concatenated input signals denoted (ND, Reg)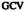 or (ND, Reg)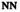. Because this involves doubling the length of the input ND signal, we compare performance of the two ILR methods with a PE NN for which the input vector is the noisy signal concatenated with itself, deemed (ND, ND).

Although our primary interest is in PE, the performance of the $\lambda_{\text{NN}}$ is of independent interest, given ongoing research in λ selection for TR. Therefore, in Section 4, we use an oracle $\lambda_{oracle}$ as a comparison standard for evaluating the performance of GCV and $\lambda_{\text{NN}}$ for regularizer selection on simulated data. We then compare five methods of PE, focusing on MWF; these are three NNs, with subscripts indicating the method of λ selection: (i) (ND, Reg)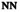, (ii) (ND, Reg)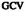, and (iii) (ND, ND) as described above; (iv) Tikhonov‐regularized Levenberg–Marquart NLLS (TR‐NLLS), using $\lambda_{\text{GCV}}$; and (v) conventional NLLS. These are all compared with TR‐NLLS using $\lambda_{oracle}$. We then present results for in vivo human brain. In this case, there is no true oracle standard of comparison, so we establish a high‐quality MWF map as a reference standard. Of note is that we apply ILR only to pixels for which the decay signal is of biexponential character. We compare the performance of the two ILR PE NNs, using, respectively, $\lambda_{\text{NN}}$ and $\lambda_{\text{GCV}}$, finding that the latter exhibits somewhat superior performance, and then compare this with the conventional (ND, ND) PE NN, not using ILR. For the convenience of the reader, we include a glossary of abbreviations in Table 1.

**TABLE 1:** Glossary of abbreviations used in the paper.

Term	Abbreviation
Input layer regularization	ILR
Noisy decay	ND
Regularized signal	Reg
Parameter estimation	PE
Neural network	NN
Nonlinear least squares	NLLS
Tikhonov‐regularized nonlinear least squares	TR‐NLLS
Generalized cross‐validation	GCV
Myelin water fraction	MWF
Minimum mean squared estimator	MMSE

In Section 5, we highlight additional aspects of the underlying problem and certain limitations of our approach.

## 2 Mathematical Formalism

### 2.1 Biexponential Signal Analysis

We assume that s(t) is measured at a finite number of echo times ${\left\{t_{n}\right\}}_{n=1}^{N}$; Equation (2) becomes

(3) $$\text{G(p)}\equiv {\left(c_{1}e^{\frac{-t_{n}}{T_{2,1}}}+(1-c_{1})e^{\frac{-t_{n}}{T_{2,2}}}\right)}_{n=1}^{N},$$

where p is the set of parameters $\left(c_{1},T_{2,1},T_{2,2}\right)$. We will further assume $t_{1}=0$ as is usual for biexponential analysis, although for the conventional MRI experiment data acquisition is initiated at a minimum echo time $TE_{0}>0$ which depends primarily on gradient and pulse duration. Additionally, **G(p)** is invariably corrupted by noise. In this paper we will address the conventional case of MR magnitude imaging, in which the noise model is Rician so that

(4) $$\text{s}=\sqrt{{\left(\text{G(p)}+\xi \right)}^{2}+\eta^{2}}$$

Here, ξ and η are Gaussian random variables of zero mean and equal variance. When the signal **s** is normalized to unity, the corresponding signal‐to‐noise ratio (SNR) will be expressed as the standard deviation σ of these random variables. In other applications, zero‐mean additive Gaussian random noise will be the most common noise model. We denote the true underlying parameter set producing the signal G as ${\text{p}}_{true}$. The goal of the analysis is to recover the parameter vector ${\text{p}}_{true}$ from the noisy measurement vector s.

### 2.2 Parameter Estimation

A straightforward way to recover $p_{true}$ from s is NLLS analysis. We note that for Rician data, this deviates from statistically validated maximum likelihood estimator analysis [32, 33] but is nevertheless conventional and accurate for reasonably high SNR. The NLLS approach involves computing an estimate ${\text{p}}^{*}$ that minimizes the mean squared difference between the model G(p) and the signal s for p∈F, where F is a feasible set of parameters. We can tailor F based on our problem, in this case constraining $T_{2,1},T_{2,2}\geq 0$ and $0\leq c_{1}\leq 1$. Therefore, the NLLS approach to PE is mathematically formulated as follows:

(5) $${\text{p}}^{*}\equiv {\text{argmin}}_{\text{p}\in F}{\;\|\text{G(p)}-\text{s}\|}_{2}^{2}$$

(6) $$=\underset{T_{2,1},T_{2,2}\geq 0,0\leq c_{1}\leq 1}{\text{argmin}}\left[\overset{N}{\underset{n=1}{\sum }}{\left(c_{1}e^{\frac{-t_{n}}{T_{2,1}}}+(1-c_{1})e^{\frac{-t_{n}}{T_{2,2}}}-s_{n}\right)}^{2}\right].$$

However, as stated, the inverse problem (5) is highly ill‐posed in the sense of Hadamard [34], meaning that distinct parameter choices $p_{1}$ and $p_{2}$ can map to nearly identical biexponential decay curves [35]. This ill‐posedness results in a high sensitivity to noise of derived parameter estimates. Stabilization of the PE problem is therefore a major topic of investigation in inverse problems.

### 2.3 Regularization

A well‐known approach to address the ill‐posedness of the problem Equation (5) is *regularization*, in which (5) is modified through the introduction of an additional term to the objective function that penalizes the complexity of feasible solutions p [36]. Among the many ways of quantifying complexity, an effective approach is to supplement (5) with TR [37], where the complexity is described through the $L^{2}$ norm of the parameters weighted against the fidelity term through the *hyperparameter* λ:

(7) $${\text{p}}_{\lambda (\text{s})}^{*}:=\underset{\text{p}\in F}{\text{argmin}}\|\text{G(p)}-\text{s}{\|}_{2}^{2}+\lambda \|B\text{p}{\|}^{2}.$$

Here, B is a 3×3 diagonal matrix:

(8) B=diag(100,1,1),

taken to equalize the scales between different parameters, because the $T_{2,1},T_{2,2}$ values are two orders of magnitude larger than $c_{1}$. Along with other variational regularization techniques such as Lasso ($L^{1}$ regularization) or elastic net (a linear combination of both $L^{2}$ and $L^{1}$ regularizers), TR effectively serves to stabilize ill‐posed inverse problems. In particular, introduction of the parameter λ≥0 improves the condition number of the PE problem [38, 39] that is, the map of the noisy signal s to the parameter set ${\text{p}}_{\lambda }^{*}(\text{s})$, as explicitly outlined for this type of system in [39]. We will henceforth term this the *TR‐NLLS* estimate. In a Bayesian framework, it provides Gaussian priors on the parameters where the TR‐NLLS estimate is the maximum a posteriori (MAP) estimator. In the present case, there is no straightforward statistical or physical interpretation of the TR‐NLLS estimator ${\text{p}}_{\lambda }^{*}(\text{s})$ and the application of TR for PE in Rician‐distributed signals is highly nonstandard. However, even lacking a true physical basis, regularization applied in (7) TR‐NLLS would be expected to improve stability.

### 2.4 Regularization Parameter Selection

The appropriate selection of λ, which controls the bias‐variance tradeoff in estimating ${\text{p}}_{true}$ through TR, is a central challenge in inverse problems. Large values lead to simple, low complexity solutions (low variance) but with low fidelity to the underlying signal s (high bias) and highly biased parameter estimates. Conversely, small values ensure the closest fidelity to the signal (low bias) but high variability to small perturbations in the signal (high variance), such as due to noise. In general, the problem of choosing the optimal λ refers to selecting the λ for which the associated solution to (7) ${\text{p}}_{\lambda }^{*}(s)$ is the closest to the true underlying parameter ${\text{p}}_{true}$. This can be mathematically formulated through the following *bilevel* optimization problem:

(9) $$\lambda_{oracle}(\text{s})=\underset{\lambda }{\text{argmin}}\|{\text{p}}_{true}-{\text{p}}_{\lambda }^{*}(\text{s})\|,\;\text{w.r.t}$$

(10) $${\text{p}}_{\lambda }^{*}(\text{s})=\underset{\text{p}\in F}{\text{argmin}}{\left\|\text{G(p)}-\text{s}\right\|}_{2}^{2}+\lambda \|\text{p}{\|}_{2}^{2}.$$

Here, (9) is referred to as the *upper level* problem, and (10) is referred to as the *lower level* problem. Note that the solution to the upper level problem depends upon the noisy signal s which in turn depends on the map G and the SNR. Thus, the bilevel formulation identifies two sources which affect the optimal λ. We term the optimal λ(s) from (9) as $\lambda_{oracle}$ and refer to the following problem as the λ− *selection problem*:

(11) $$\text{Recover}\;\lambda_{oracle}\;\text{from}\;\text{s}$$

Typically, it is infeasible to directly solve the bilevel problem (9) and (10) because ${\text{p}}_{true}$ is not available in applications. Moreover, establishing theoretical guarantees for the existence and positivity of solutions to the bilevel problem (9) and (10) is an active area of research [40]. Consequently, a great deal of theory is dedicated to approximating $\lambda_{oracle}$ using alternative formulations and algorithmic techniques [41, 42]. In practice, numerical methods for choosing λ are often implemented, including the L‐curve (LC) [43], discrepancy principle (MDP) [44], or GCV [14]. Among these, GCV stands out as a theoretically and practically robust method for approximating $\lambda_{oracle}$ [45, 46, 47].

### 2.5 Neural Networks for Parameter Estimation

A fully connected L‐layer NN is defined by the alternating composition of linearities and nonlinearities:

(12) $$f_{\Theta }(x)=W_{L}\left(\sigma (W_{L-1}(\text{…}x))+b_{L-1}\right)+b_{L}$$

Given a training set consisting of input‐output pairs ${\left\{x_{i},y_{i}\right\}}_{i=1}^{m}$, the network weights, $W_{i}$, and biases, $b_{i}$, where we write $\Theta ={\left\{W_{i},b_{i}\right\}}_{i=1}^{L}$, are determined by optimizing a loss function ℒ measuring the discrepancy between the true outputs $y_{i}$ and the NN outputs $f(x_{i})$:

(13) $$\Theta =\underset{\theta }{\text{argmin}}\frac{1}{m}\overset{m}{\underset{i=1}{\sum }}ℒ(y_{i},f_{\theta }(x_{i}))$$

NNs are well established in the setting of inverse problems [48] in the biomedical sciences and offer a model‐free approach to mapping an unseen signal to parameter estimates. Unlike classical mapping methods (such as TR‐NLLS), NNs require training with synthetically or experimentally generated data. Such approaches involving NNs routinely outperform classical methods as long as the underlying NN is well trained [49, 50, 51]. In fact, NNs and classical methods like TR‐NLLS may be combined. For example, in [13], a supervised learning approach to estimating $\lambda_{oracle}$ was presented, wherein signals s are generated from a known underlying $p_{true}$, followed by direct calculation of the corresponding optimal $\lambda ,\;\lambda_{oracle}$, leading to training pairs of the form $\left\{\text{s},\lambda_{oracle}(\text{s})\right\}$. A neural network can then be trained on this data and serve as a surrogate mapping for $\lambda_{oracle}$. We will elaborate on this process in Section 3. These considerations allow us to define two distinct NNs for the present problem:

1. NNs for λ‐selection: Following [13], we develop an NN, termed $\lambda_{\text{NN}}$, for λ selection. We compare this with $\lambda_{\text{GCV}}$ as defined in Section 2.4.
2. NNs for PE: We develop a separate NN for estimations of the parameter ${\text{p}}_{true}$ from the noisy signal ${\text{s}}_{i}$.

### 2.6 Input Layer Regularization

In this paper, we combine λ‐selection with an NN for PE into a pipeline for estimating ${\text{p}}_{true}$. The resulting architecture, which we call *ILR* [12], is defined by concatenation of the noisy signal data (ND) s with, or augmented by, a regularized (Reg) version given by $G\left({\text{p}}_{\lambda (\text{s})}^{*}\right)$ where ${\text{p}}_{\lambda (\text{s})}^{*}$ is a TR‐NLLS parameter estimate from (7) with hyperparameter λ(s) chosen through a user‐defined λ‐selection method. The augmented input vector is thus given by

(14) $$\text{x}:=\left[\text{s},\text{G}({\text{p}}_{\lambda (\text{s})}^{*}(\text{s}))\right].$$

An NN (termed (ND, Reg)) is trained to estimate ${\text{p}}_{true}$ from x (see Figure 1).

#### 2.6.1 Theoretical Justifications for ILR

ILR may be conceived as a deep learning–based instantiation of the empirical Bayes estimator of p [52]. To make this connection precise, first consider the minimum mean squared estimator (MMSE) of p from s, given by 𝔼[p|s], the conditional expectation of p given s. Moreover, suppose we had a *prior* for p, depending on hyperparameters λ with the MMSE 𝔼[p|s,λ]. Note that mean squared errors for both MMSEs (with prior and without prior) can be given in terms of the variances of the conditional distributions of p:

(15) $$\text{MSE}(𝔼[p|s]):={𝔼}_{p,s}[\|p-𝔼[p|s]{\|}_{2}^{2}]={𝔼}_{s}[\mathrm{Var}(p|s)],$$

(16) $$\text{MSE}(𝔼[p|s,\lambda ]):={𝔼}_{p,s,\lambda }[\|p-𝔼[p|s,\lambda ]{\|}_{2}^{2}]={𝔼}_{s,\lambda }[\mathrm{Var}(p|s,\lambda )].$$

Moreover, note that by the law of total variance, conditioning on λ, we have

(17) $$\mathrm{Var}(p|s)={𝔼}_{\lambda }[\mathrm{Var}(p|s,\lambda )]+{\mathrm{Var}}_{\lambda }(𝔼[p|s,\lambda ]|s).$$

Taking expectations over the signal s, we get

(18) $$\text{MSE}(𝔼[p|s])=\text{MSE}(𝔼[p|s,\lambda ])+{𝔼}_{s}[\mathrm{Var}(𝔼[p|s,\lambda ]|s)].$$

This proves that under *full knowledge* of the hyperparameters λ, the MMSE that uses prior information does better than the MMSE that does not. In practice, however, we do not have access to the prior. In this case, under the empirical Bayes scheme, we would compute a point estimator λ(s) using the data s and then obtain an estimator for p given by μ(s,λ(s)). Using a decomposition p−μ=(p−𝔼[p|s,λ])+(𝔼[p|s,λ]−μ), we have

(19) $$\text{MSE}(\mu (s,\lambda (s)):=𝔼[\|p-\mu (s,\lambda (s)){\|}_{2}^{2}]$$

(20) $$=\text{MSE}(𝔼(p|s,\lambda ))+𝔼[\|\mu (s,\lambda )-𝔼[p|s,\lambda ]{\|}_{2}^{2}].$$

Combining this decomposition with (18), we have

(21) $$\text{MSE}(\mu )=\underbrace{\text{MSE}(𝔼[p|s])-{𝔼}_{s}[\mathrm{Var}(𝔼[p|s,\lambda ]|s)]}$$

(22) $$+𝔼[\|\mu (s,\lambda )-𝔼[p|s,\lambda ]{\|}_{2}^{2}].$$

The first term grouped under the bracket is *fundamental*, and it is strictly smaller than the MMSE of p without a prior. The term that contributes to the error is the proximity of the estimator μ to the MMSE with prior. Therefore, it is beneficial to design an estimator λ(s) and an estimator μ depending on both the signal s and λ(s) that is as accurate as possible. This is what we attempt to do in ILR with λ‐selection. In particular, we make the following modeling assumptions:

(23) s∼ℙ(s|p),p∼ℙ(p|λ)

Under this setup, a neural network trained to carry s to p would be surrogate for 𝔼[p|s] and the proposed estimator μ(s,λ(s)) is our ILR network trained to carry a point estimator of λ and the signal s to p via concatenation. Here, the point estimator is selected via the bilevel optimization problem (9) and (10) and is pushed into an estimator, that is, the (ND, Reg) network such that the MMSE‐empirical Bayes error in (22) is minimized. However, our framework deviates from the empirical Bayes in two crucial ways: (1) $\lambda_{oracle}$ is not necessarily optimal in a statistical framework (like a MAP estimator, for instance), and (2) given Rician noise in s the lower level program (10) is not a maximum likelihood estimator of p. Despite these estimators not having a direct statistical interpretation, they will nevertheless be maximally informative of p.

Another significant deviation from empirical Bayes arises from the fact that $\lambda_{oracle}$ uses $p_{true}$; as such, this scheme would be could not be implemented in practice at the inference step of the analysis when one does not have access to the underlying true parameter. However, by realizing that we can generate instances of $p_{true}$ and $\lambda_{oracle}$, a neural network can be trained to approximate a point estimator of λ. Therefore, at the inference step, we may simply use the observed signal s to obtain λ(s).

## 3 Methods

**FIGURE 2:**
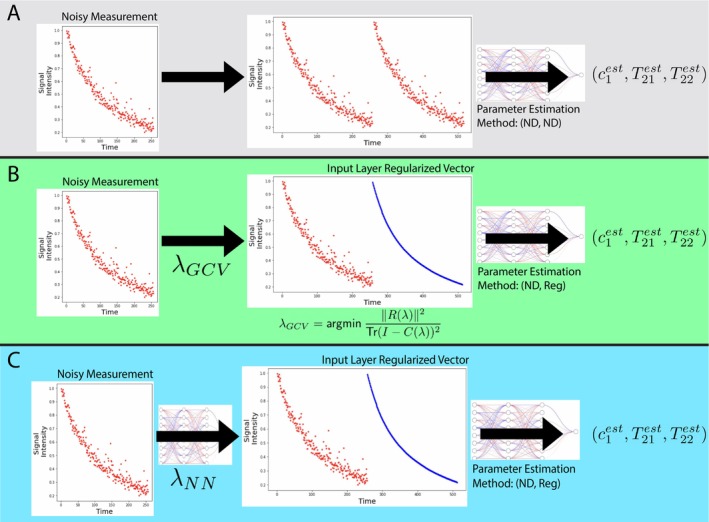
We compare the three approaches of parameter estimation. (A) The conventional method of estimating parameters from the noisy decay (ND) itself. (B,C) A novel approach for parameter estimation, where the first step involves selecting either GCV or NN for estimating λ, labeled $\lambda_{\text{GCV}}$ and $\lambda_{\text{NN}}$, respectively. Training data for the ILR networks (ND, Reg) are first passed through a TR‐NLLS solver with the signal‐dependent λ(s) to construct the concatenated vector x in Equation (14). C(λ) is defined according to Equation (28).

As above, we propose a methodology combining ILR with signal‐based selection of λ for solving the PE problem. We compare two choices for generating λ: (1) a neural network, following [13], and (2) GCV (outlined in Section 3.2). See Figure 2. In Section 4.1, we discuss the choice between use of $\lambda_{\text{NN}}$ and $\lambda_{\text{GCV}}$.

We now outline our algorithm for each choice of estimating λ.

### 3.1 Selecting λ With an NN Followed by Parameter Estimation

To estimate λ by an NN, $\lambda (\text{s}):=\lambda_{\text{NN}}(\text{s})$, we train $\lambda_{\text{NN}}$ using synthetically generated training data $\left\{{\text{s}}_{i},\lambda_{oracle}({\text{s}}_{i})\right\}$. Given $\text{s},\;\lambda_{oracle}$ is found using the Brent method which is a combination of golden‐section search and parabolic interpolation, across the search space $\left[10^{-7},10^{3}\right]$, optimizing (9). We summarize the data generation process in Appendix A. Here, $\lambda_{\text{NN}}$ is a convolutional neural network (CNN) with the architecture shown in Figure A1. We make use of $L^{1}$ loss to train the NN to recognize $\lambda_{oracle}$ in order not to heavily penalize outliers because the distribution of $\lambda_{oracle}$ spans several orders of magnitude (Figure 4). Thus, the loss function used for training $\lambda_{\text{NN}}$ is given by

(24) $${ℒ}_{\lambda }:=\frac{1}{N}\overset{N}{\underset{i=1}{\sum }}|\lambda_{oracle}({\text{s}}_{i})-\lambda_{\text{NN}}({\text{s}}_{i})|$$

We provide model architecture and training details in Appendix B.

To recover parameter estimates, we generate the concatenated input ${\text{x}}_{i}$ according to (14) with $\lambda_{\text{NN}}(\text{s})$ in place of λ(s). Letting ${\text{p}}_{i}$ be the parameter vector used for generating the noisy signal ${\text{s}}_{i}$, we train a neural network (ND, Reg)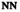 for recovering ${\text{p}}_{i}$ from ${\text{x}}_{i}$ by minimizing mean squared error. The loss function for training (ND, Reg)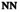 is given by

(25) $$ℒ:=\frac{1}{N}\overset{N}{\underset{i=1}{\sum }}\|{\text{p}}_{i}-{\text{(ND, Reg)}}_{\text{NN}}\left({\text{x}}_{i}\right){\|}_{B}^{2}$$

Here, $\|\cdot {\|}_{B}^{2}$ is the *weighted* mean squared error norm given by $\|x{\|}_{B}^{2}=x^{⊤}Bx$, where B is the diagonal matrix (8).

### 3.2 Selecting λ With GCV Followed by Parameter Estimation

Here, we describe the algorithm used for producing $\lambda_{\text{GCV}}(\text{s})$. Recall that G in (3) maps a parameter combination $\text{p}\in {\mathbb{R}}^{3}$ to a noiseless signal vector $\text{G}(\text{p})\in {\mathbb{R}}^{N}$. G is a differentiable map from ${\mathbb{R}}^{3}$ to ${\mathbb{R}}^{N}$ and admits a Jacobian $D\text{G}:{\mathbb{R}}^{3}\to {\mathbb{R}}^{N}$. Next, recall that for a fixed $\lambda ,\;{\text{p}}_{\lambda }^{*}(\text{s})$ is the TR‐NLLS parameter estimate (7). This allows us to define a Jacobian DG, a matrix‐valued function that depends *only* on λ:

(26) $$J(\lambda ):=D\text{G}({\text{p}}_{\lambda }^{*}(\text{s}))$$

Then, $\lambda_{\text{GCV}}$ for nonlinear inverse problems is defined as the minimizer of a function involving J(λ) [47]:

(27) $$\lambda_{\text{GCV}}(\text{s})=\underset{\lambda \geq 0}{\text{argmin}}\;\text{GCV}(\lambda )$$

(28) $$:=\underset{\lambda \geq 0}{\text{argmin}}\;\frac{\|G\left({\text{p}}_{\lambda }^{*}\left(\text{s}\right)\right)-{\text{s}}_{i}{\|}_{2}^{2}}{\text{Tr}\left[{\left(I-J(\lambda ){\left(J{\left(\lambda \right)}^{⊤}J(\lambda )+\lambda I\right)}^{-1}J{\left(\lambda \right)}^{⊤}\right)}^{2}\right]}$$

The concatenated ILR input vectors are produced according to (14) with $\lambda_{\text{GCV}}(\text{s})$ used place of λ(s). The resulting NN trained to minimize the loss (25) is termed (ND, Reg)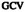 (Figure 2). Note that constructing training data for either the NN or GCV‐based approach requires grid‐based optimization for obtaining $\lambda_{oracle}({\text{s}}_{i})$. However, once training is completed, $\lambda_{\text{NN}}$ requires no further grid‐based searches for operation, while obtaining $\lambda_{\text{GCV}}({\text{s}}_{i})$ still requires this at the testing, or implementation, step, in order to solve (28).

## 4 Results

### 4.1 Parameter Estimation

#### 4.1.1 Estimating $\lambda_{oracle}$

Learning the signal‐dependent optimal TR parameter $\lambda_{oracle}$ is a problem of independent interest in inverse problems. Here, we present our results for estimation of the optimal λ for the three‐parameter problem Equation (5).

**FIGURE 3:**
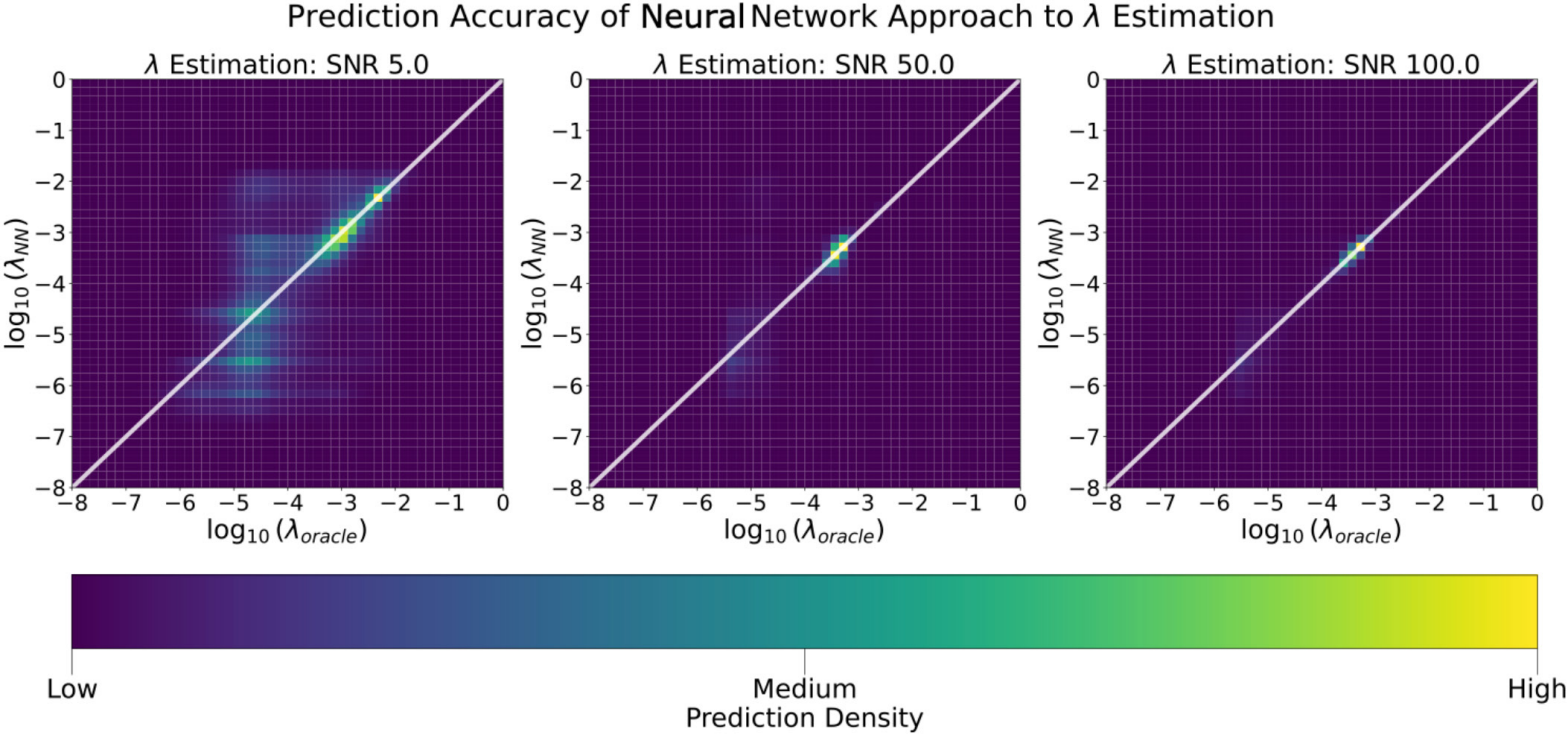
3D Histogram showing prediction density of the λ‐selection neural network versus the $\lambda_{\text{oracle}}$ approach detailed in Algorithm A1. The white line represents the y=x line, indicating the benchmark for perfect prediction. Left to right: SNRs 5 (low), 50 (medium), and 100 (high).

Figure 3 shows that the λ‐selection network $\lambda_{\text{NN}}$ when trained on the $L^{1}$ loss for solving the bilevel problem (9) closely approximates $\lambda_{oracle}$ over unseen examples of noisy signals s. As expected, estimation quality improves with values clustering more closely to the diagonal as SNR increases.

In Table 2, we compare $\lambda_{\text{NN}}$ and $\lambda_{\text{GCV}}$ in terms of reproducing the distribution of the values of $\lambda_{oracle}$ as quantified by the *earth mover's distance*, also known as the Wasserstein‐1 distance. We find that the distribution of the ensemble $\left\{\lambda_{\text{NN}}(\text{s})\right\}$ is closer to the true ensemble than the distribution of $\left\{\lambda_{\text{GCV}}(\text{s})\right\}$ (Table 2).

**TABLE 2:** The Wasserstein‐1, or earth mover's distance (EMD), between the ensembles of λ values produced by the NN (upper row) and GCV (lower row).

SNR	5.0	50.0	100.0
$W_{1}(\lambda_{\text{NN}}(\text{s}),\lambda_{oracle}(\text{s}))$	0.348	0.246	0.145
$W_{1}(\lambda_{\text{GCV}}(\text{s}),\lambda_{oracle}(\text{s}))$	1.386	0.862	0.560

*Note:* The NN outperforms GCV in matching the distribution to $\lambda_{oracle}$ for every SNR level. However, the multiple modes towards low values of λ are captured more accurately by GCV.

**FIGURE 4:**
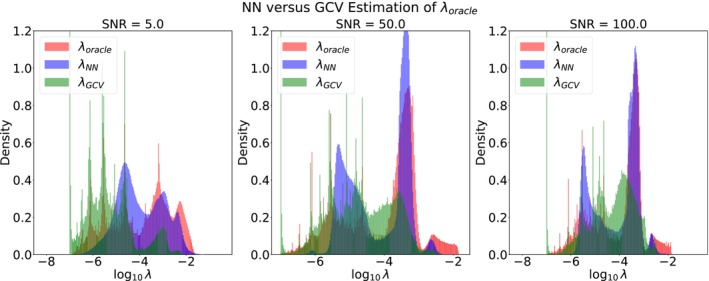
Left to right: The true λ distribution (orange) overlaid with the distributions of $\lambda_{\text{NN}}$ and $\lambda_{\text{GCV}}$. The true distributions at the low SNR level are more multimodal and have greater spread, with GCV providing superior estimates as compared with NN λ selection. This is particularly seen at lower values of λ, where little‐to‐no regularization is needed. At the higher SNRs, the optimal λ values are concentrated around a small number of modes, with approximation of $\lambda_{oracle}$ by the NN now exhibiting performance superior to GCV.

In Figure 4, we disaggregate the values in Table 2 by comparing the ensembles of $\left\{\lambda_{\text{NN}}(s)\},\{\lambda_{\text{GCV}}(s)\right\}$, and $\left\{\lambda_{oracle}(s)\right\}$ values over the testing set. Despite $\lambda_{\text{NN}}$ outperforming $\lambda_{\text{GCV}}$ according to the $W_{1}$ distance overall, $\lambda_{\text{GCV}}$ replicates the distribution of $\lambda_{oracle}$ more faithfully in the regime of low SNR and small $\lambda ,\;\lambda \in [10^{-7},10^{-4}]$. These results can be described by histogram multimodality and spread in this regime, where the $\lambda_{oracle}$ distribution has greater spread and exhibits multimodality that is more accurately captured by GCV than by the NN λ estimation, which tends to provide a single dominating mode for these lower values of λ (left panel, Figure 4). Thus, although the median $\lambda_{oracle}$ is larger for low SNR as expected, the strategy for estimating $\lambda_{oracle}$ accurately over the ensemble of noisy signals s depends on higher order statistics of the $\lambda_{oracle}$ distribution. This suggests that in real applications, an initial noise‐estimation step may be of benefit when deciding between use of $\lambda_{\text{GCV}}$ and $\lambda_{\text{NN}}$.

#### 4.1.2 Accuracy of ILR and Alternative Methods for $c_{1}$ Estimation

We have defined the two ILR NN, (ND, Reg)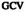 and (ND, Reg)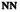, above. For comparison with conventional NN estimation, we duplicate the native noisy input vector to establish an input of the same length as the ILR NN's and thereby eliminate input signal length as a potential confounder. We call this NN (ND, ND). Additional standards of comparison are the conventional NLLS estimation method, as well as the TR‐NLLS approach in which regularization is applied to this PE problem; in our case, we have implemented this with selection of λ via GCV.

Results for estimation of $c_{1}$, representing MWF in our motivating application, are shown in Table 3. Several points of interest are evident. First, (ND, Reg)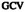 and (ND, Reg)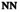 perform essentially indistinguishably. However, both of these ILR‐based NN outperform the NN without ILR, (ND, ND), across all SNR levels. This is our main result. We also note (not shown) that the NN architecture without ILR and without duplication of the input vector, deemed (ND), performs virtually identically with (ND, ND). Further, the most conventional analysis, NLLS, exhibits performance substantially inferior to any of the NN's. In addition, the nonconventional method of TR‐NLLS, in spite of the introduction of bias through regularization, provides sufficient stabilization that its overall performance is markedly superior to that of NLLS. In Appendix B, we provide a more detailed study of (ND, Reg)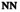 and (ND, Reg)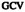 by disaggregating the RMSE over all parameter combinations appearing in the testing data.

**TABLE 3:** Root mean squared error (RMSE) in $c_{1}$ for each indicated parameter estimation method.

SNR	5.0	50.0	100.0
(ND, Reg) 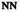 ${\text{RMSE}}_{c_{1}}$	0.1652	0.0931^a^	0.0796^a^
(ND, Reg) 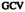 ${\text{RMSE}}_{c_{1}}$	0.1643^a^	0.0945	0.0817
(ND, ND) ${\text{RMSE}}_{c_{1}}$	0.1671	0.1079	0.0935
TR‐NLLS ${\text{RMSE}}_{c_{1}}$, $\lambda_{oracle}$	0.2328	0.2092	0.2078
TR‐NLLS ${\text{RMSE}}_{c_{1}}$, $\lambda_{\text{GCV}}$	0.3687	0.3487	0.3347
NLLS ${\text{RMSE}}_{c_{1}}$	0.3844	0.3471	0.3453

^a^ Superior performance for the given SNR category.

### 4.2 MWF Estimation

We now apply ILR to the estimation of the MWF from magnetic resonance relaxometry (MRR) signals from the human brain. A multispin‐echo sequence with 64 values of TE was applied as outlined in Appendix A, with the sampling scheme leading to the decay signal as described in Equation (2).

However, we note that while the white matter of the brain, rich in myelin content, is expected to exhibit biexponential decay, the gray matter, with little‐to‐no myelin, is more likely to be better described by a monoexponential decay. For the latter, Equation (2) represents an underdetermined model, and no amount of regularization or other manipulation will permit meaningful parameter values to be extracted. The determination of two exponentials will be enforced by the model, with their fractions $c_{1}$ and $c_{2}$ noise dependent and therefore random. Therefore, we wish to apply our analysis only to truly biexponential signals. Accordingly, we evaluate each pixel using the AIC [53] and analyze signals only from pixels that are better described as biexponential rather than monoexponential. We note that the distribution of the AIC‐defined monoexponential and biexponential pixels closely follows the general pattern of cortical gray and interior white matter in the brain.

#### 4.2.1 Benchmarking

**FIGURE 5:**
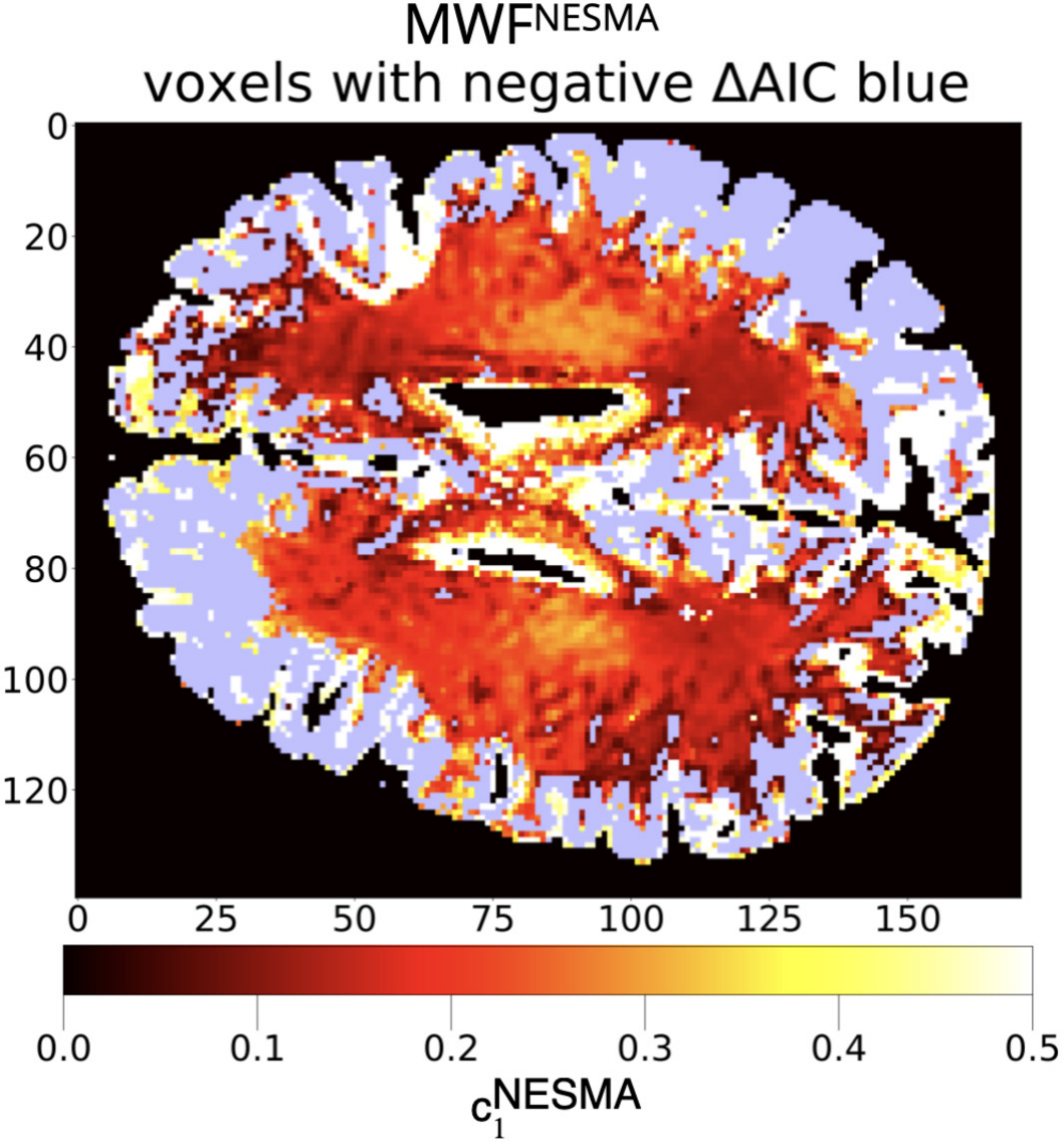
Comparison standard for $c_{1}$ for pixels determined to be biexponential according to the AIC (color bar); purple pixels are those determined to be of a monoexponential character. Each voxel in the 288×288 image of the brain is characterized by a noisy signal s with 64 acquisition times. Each of these voxel signals was evaluated according to a five‐parameter model after NESMA filtering [54]. Estimates for $c_{1}$ were taken as a comparison standard.

With this, the time series for each voxel under consideration is a single noisy biexponential curve with parameters to be estimated via ILR with signal‐dependent λ selection. To create a reference set of parameters, we used nonlocal estimation of multispectral magnitudes (NESMA) denoising filter [54], followed by five‐parameter NLLS estimation. We include additional information regarding the acquisition of brain data in Appendix E. The NESMA estimates for $c_{1}$ are visualized in Figure 5. We recognize that the NESMA‐filtered NLLS parameters are limited in reflecting the true underlying parameters; therefore, the simulation data may be more indicative of model performance on the biexponential signal in this sense.

We also benchmark our method against NLLS, TR‐NLLS with $\lambda_{\text{GCV}}$, and the deep learning method known as MI‐ML [30]. In MI‐ML, a neural network trained to recover $p_{true}$ from a loss given by a combination of MSE and Wasserstein‐1 loss, which compares the distance between $p_{est}$ and $p_{true}$ when viewed as distributions. We provide further details on MI‐ML in Appendix E.4. We retrain both (ND,ND) and (ND, Reg) models with this loss function.

As suggested in simulations, high‐quality performance of the (ND, Reg) networks is predicated on proper selection of the regularization parameter λ using either GCV or NN; in our case, this means selection of λ that is similar to $\lambda_{oracle}$ or at least that provides comparable performance. Furthermore, we illustrated that the choice of GCV or NN can be made based on estimation of SNR. Using a comparable method of SNR quantification, we found median SNR of ≈25.0 on the biexponential pixels identified by the AIC, suggesting that the underlying distribution of $\lambda_{oracle}$ was likely to be spread over orders of magnitudes with a large number of modes, as in Figure 4. Table 3 indicates that superior results may be obtained from (ND, Reg)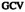 as compared with (ND, Reg)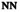.

**FIGURE 6:**
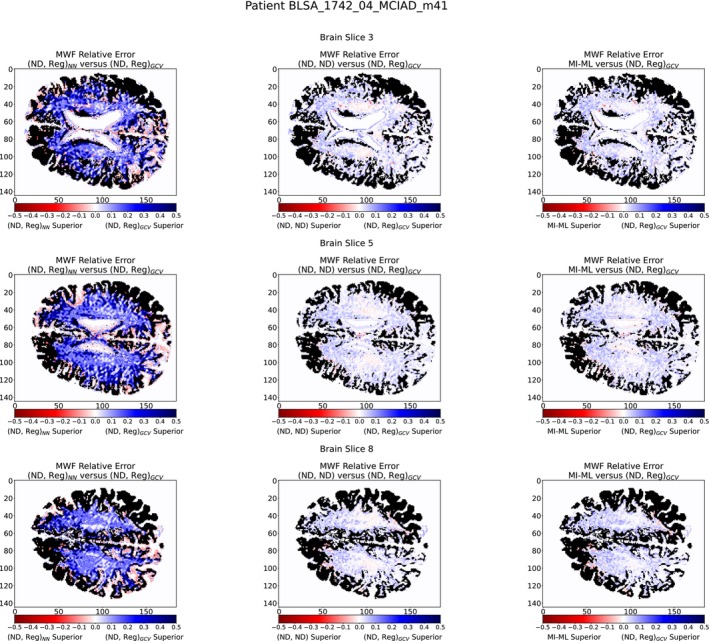
Comparison of $c_{1}$ estimation according to the two methods of λ selection and the MI‐ML benchmarking model. The metric illustrated is the difference in absolute values of $\left|Metho{d_{1}}_{c_{1}}^{est}-c_{1}^{NESMA}|-|Metho{d_{2}}_{c_{1}}^{est}-c_{1}^{NESMA}\right|$. For example, the left column is computed via $\left|({\text{(ND, Reg)}}_{\text{NN}}^{c_{1}^{est}})-c_{1}^{NESMA}|-|({\text{(ND, Reg)}}_{\text{GCV}}^{c_{1}^{est}})-c_{1}^{NESMA}\right|$ calculated over pixels with a biexponential character. As seen, ${\text{(ND, Reg)}}_{\text{GCV}}$ is superior overall.

**FIGURE 7:**
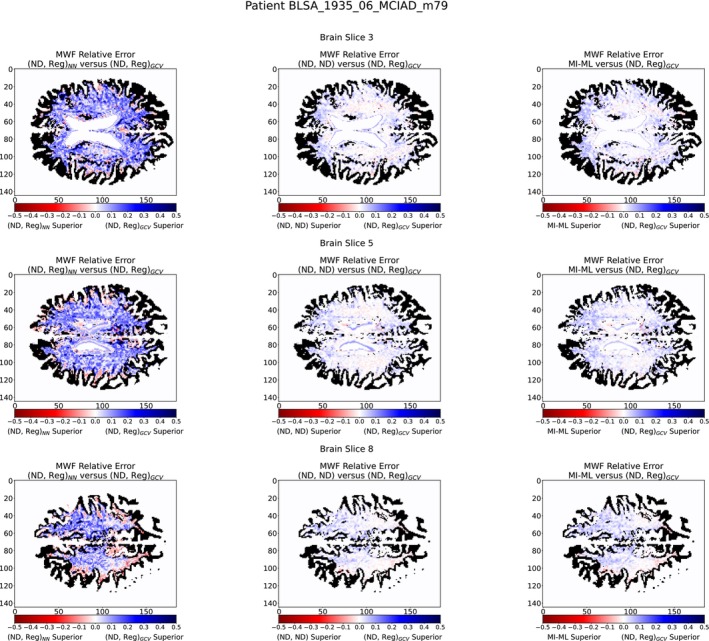
Comparison of $c_{1}$ estimation according to the two methods of λ selection and the MI‐ML benchmarking model. The metric illustrated is the difference in absolute values of $\left|Metho{d_{1}}_{c_{1}}^{est}-c_{1}^{NESMA}|-|Metho{d_{2}}_{c_{1}}^{est}-c_{1}^{NESMA}\right|$. For example, the left column is computed via $\left|({\text{(ND, Reg)}}_{\text{NN}}^{c_{1}^{est}})-c_{1}^{NESMA}|-|({\text{(ND, Reg)}}_{\text{GCV}}^{c_{1}^{est}})-c_{1}^{NESMA}\right|$ calculated over pixels with a biexponential character. As seen, ${\text{(ND, Reg)}}_{\text{GCV}}$ is superior overall.

Indeed, the left column of Figures 6 and 7 show that (ND, Reg)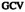 is in general superior to (ND, Reg)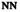. This is also consistent with Figure 4, suggesting that $\lambda_{\text{GCV}}$ may more accurately reproduce the modes of the distribution of $\lambda_{oracle}$; this capability is not captured by either the $L^{1}$ loss or the earth mover's distance. Therefore, $\lambda_{\text{GCV}}$ may be preferable for use with ILR with limited SNR.

Adopting (ND, Reg)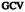 as the method of choice for this application, we compared its performance with that of the (ND, ND) network, as shown in the middle column of Figures 6 and 7. We find that (ND, Reg)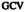 outperforms (ND, ND) nearly *everywhere*.

We further expand our significance by benchmarking our best performing method to the MI‐ML model as described in Section 1.2. We see that (ND, Reg)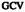 performs superior to MI‐ML across almost all pixels, as shown in the right column of Figures 6 and 7. The summary of root mean squared deviation from ${MWF}^{NESMA}$ are reported in Table 4, with (ND, Reg)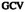 performing superiorly for all slices across all patients. From Table 5, it is evident that our best performing method is a significant gain over alternative methods. Together, these results indicate the efficacy of ILR to enhance NN‐based MWF estimation. It is notable that optimal performance requires combining this PE NN with a classical technique for selection of the regularization parameter $\lambda ,\;\lambda_{\text{GCV}}$, rather than selection according to the $\lambda_{\text{NN}}$, for generating the augmented part of the noisy input signal.

**TABLE 4:** Root mean squared deviation from $MWF^{NESMA}$ in MWF for each parameter estimation method.

	Root mean squared deviation from $MWF^{NESMA}$						
Brain slice	(ND, ND)	(ND, Reg) 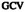	(ND, Reg) 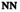	NLLS	MI‐ML	${\text{MI‐ML (ND, Reg)}}_{\text{GCV}}$	${\text{MI‐ML (ND, Reg)}}_{\text{NN}}$
m41‐3	0.1277	**0.1147**	0.1818	0.2462	0.1273	0.1264	0.2348
m41‐5	0.0744	**0.0673**	0.1118	0.1433	0.0740	0.0757	0.1422
m41‐8	0.1004	**0.0898**	0.1449	0.2093	0.1016	0.1010	0.1966
m79‐3	0.1015	**0.0944**	0.1406	0.2206	0.1038	0.1043	0.2267
m79‐5	0.1046	**0.0973**	0.1390	0.2243	0.1066	0.1063	0.2242
m79‐8	0.0717	**0.0666**	0.0871	0.1673	0.0731	0.0733	0.1506
Aggregate	0.0967	**0.0884**	0.1342	0.2018	0.0977	0.0978	0.1959

*Note:* The bolded values represent superior performance. The aggregate row represents the averaged result over all slices and patients.

**TABLE 5:** Percentage improvement of the best performing method (ND, Reg) over the respective alternative method.

	Percentage improvement of input layer regularization over alternative methods		
Brain slice	(ND, Reg) 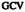 vs. NLLS	(ND, Reg) 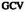 vs. (ND, ND)	(ND, Reg) 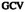 vs. MI‐ML
m41‐3	53.41%	10.18%	9.90%
m41‐5	53.04%	9.54%	9.05%
m41‐8	57.10%	10.56%	11.61%
m79‐3	57.21%	7.00%	9.06%
m79‐5	56.62%	6.98%	8.72%
m79‐8	60.20%	7.11%	8.89%
Aggregate	56.26%	8.56%	9.54%

*Note:* The aggregate row represents the averaged result over all slices and patients.

## 5 Discussion

Combining conventional analytic methodology and domain knowledge with machine learning‐based methods has become a common paradigm [55, 56, 57]. Examples include incorporating physical constraints into loss functions [58], seeding classical solvers with machine‐learned initializations [59], and designing individual layers of neural networks around the physics of the problem [60]. Here, we have presented a new approach that is an additional example of such a methodology by integrating the classical technique of TR [61, 62, 63] and bilevel optimization [64, 65] with neural network data concatenation and automated hyperparameter tuning. This leads to a refined approximation of the map from a noisy signal s to a parameter estimate $\hat{\text{p}}$. In particular, we have presented a cascaded approach to PE with two levels: (1) estimating the hyperparameter λ from the noisy signal s followed by (2) estimating the parameters of a decaying exponential using ILR, with the regularized component of the concatenated signal generated using λ from the previous step. As demonstrated in Section 2, this can be viewed as an instantiation of the empirical Bayes formulation.

For the first level of analysis, we explored two strategies for recovering λ from the noisy signal s, which can itself be viewed as a PE problem. The first strategy involves selection of lambda using GCV [14], a common, statistically validated, method that does not require an estimate of signal noise level. This approach requires no training, but rather evaluates each signal individually and estimates the optimal lambda by what is in effect a leave‐one‐out cross‐validation. The second strategy is a neural network [13] trained with a loss function involving $\lambda_{oracle}$. Both methods involve computational bottlenecks related to the solutions of optimization problems. The GCV estimate requires minimization over [0,∞) for the GCV functional, which can be difficult to optimize with gradient methods, while the NN approach requires the generation of $\lambda_{oracle}$ values through an expensive grid search strategy sweeping over several orders of magnitude. Nonetheless, we see that a four‐layer CNN can approximate the map s→λ(s) accurately, suggesting that even a modest number of layers can suffice for approximating solutions to this highly unstable problem. We have also demonstrated that the $L^{1}$ loss is an effective tool to approximate not only the map $\text{s}\to \lambda_{oracle}(\text{s})$ but also the *ensemble* of $\lambda_{oracle}$ considered as a probability distribution. The $\lambda_{\text{NN}}$ outperforms $\lambda_{\text{GCV}}$ with respect to both of these error metrics. However, we also find that at lower SNR, $\lambda_{\text{GCV}}$ appears to be superior in deriving parameters from noisy signals that do not require substantial regularization. The effect of NN depth, loss functions, and selection of activation functions on estimation of optimal λ values remains an open question.

For the second level of our analysis pipeline, we used $\lambda_{\text{GCV}}$ and $\lambda_{\text{NN}}$ to construct a regularized signal which was concatenated with the noisy signal, with this concatenated construct used to train the ILR networks (ND, Reg)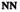 and (ND, Reg)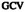. Both of these (ND, Reg) networks, differing only in the manner in which λ was selected, attained over 5%–10% or greater improvements in accuracy compared with the basic ND NN and TR‐NLLS estimation. This indicates that ILR is a promising approach for overcoming the limitations of traditional methods when performing PE from ND signals. In particular, our results suggest that ILR may significantly improve the accuracy and reliability of PE problems in MRR, a topic of great current interest for gaining insights into the pathophysiology of the CNS.

In actual brain data, there is no gold standard for PE, and surrogates for actual underlying values must be used instead. In the present case, we applied an effective noise‐reduction filter to obtain the benchmark results shown in Figure 5. Nevertheless, application to an actual in vivo system is an important part of demonstrating the potential utility of the ILR approach, especially because actual data are unlikely to strictly obey any imposed signal model. We find that the “hybridized” approach—where the λ is picked with GCV followed by NN estimation with ILR is superior to pure deep learning or pure classical estimation.

The data‐concatenation approach we have developed and implemented demonstrates a substantial improvement in the accuracy of PE for the biexponential decay signal. Nevertheless, there are certain limitations to our study which may form the basis for additional investigation. The present analysis does not incorporate the spatial correlations between pixels which exist in any natural image. Instead, our $\lambda_{\text{NN}}$ networks are trained to process data arising from individual decay curves. Thus, although $\lambda_{\text{NN}}$ provides meaningful estimates of $\lambda_{oracle}$, further optimization of its architecture, including potential modifications of the penalty function to penalize rapid spatial variation, may improve results. The challenges in this approach are to avoid the introduction of bias and of blurring.

Additionally, although we have carefully tuned the hyperparameters for our various NNs, this remains something of a black art in the literature and results could change somewhat with additional tuning. We have elected to perform our investigations using the Rician noise model as the basic model in MRI, and there is nothing in our analysis that would be specific to this choice. Nevertheless, investigation of other models, and in particular the Gaussian, would be of interest. In addition, as noted above, we have demonstrated our method on actual brain data, but these results are somewhat inferential, because there is no true gold standard for underlying parameter values. We also note that one of our comparison methods, TR‐NLLS, is highly nonstandard. We have however found this to be an effective modification of NLLS in many settings and are exploring this more systematically. Further, we applied the AIC to define pixels for which signal regularization makes sense; this appears to be a novel approach in the literature and has proved to be very effective here. However, there are several choices, including the F test, the Bayesian information criterion, and reduced chi‐squared, to address this issue, and another choice may exhibit superior performance. Lastly, we highlight the fact that our work as presented here explores only the biexponential model, with emphasis on determination of the $c_{1}$ parameter. Other signal models and PE problems should also be evaluated in this framework.

In summary, we have addressed the recovery of the parameter $c_{1}$ in the biexponential model (3) for MWF estimation in the CNS, a topic of ongoing research interest. On both simulated data and actual brain data, we find ILR to offer a significant improvement in PE accuracy.

## Author Contributions

**Mirage Modi:** conceptualization, methodology, software, validation, investigation, data curation, writing – original draft, writing – review and editing, visualization. **Shashank Sule:** conceptualization, methodology, formal analysis, validation, investigation, writing – original draft, writing – review and editing, visualization. **Jonathan Palumbo:** software, resources. **Michael Rozowski:** software, resources. **Griffin S. Hampton:** resources, data curation. **Mustapha Bouhrara:** resources, data curation. **Wojciech Czaja:** conceptualization, writing – review and editing, supervision, project administration, funding acquisition. **Richard G. Spencer:** conceptualization, writing – review and editing, supervision, project administration, funding acquisition.

## Conflicts of Interest

The authors declare no conflicts of interest.

## Figures and Tables

**FIGURE B1:**
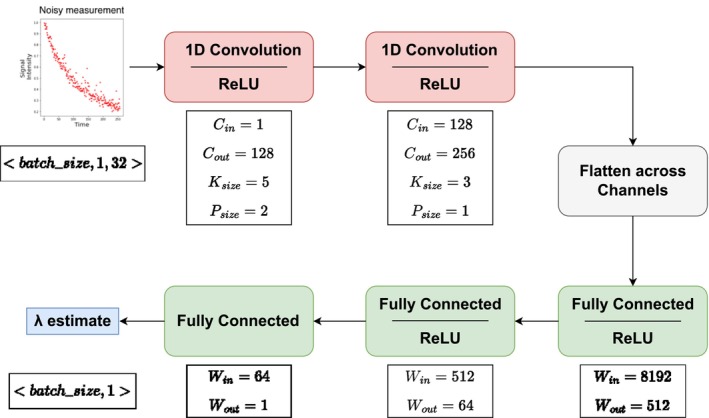
$\lambda_{\text{NN}}$ architecture. One‐dimensional convolutional layers are applied to the input noisy signal, followed by fully connected layers to predict $\lambda_{oracle}$. C refers to the channel sizes (in/out for input or output channels, respectively). We also define the kernel size as $K_{size}$ and the padding as $P_{size}$. W refers to the width of a given fully connected layer. The ReLU activation function is used between each layer.

**FIGURE D1:**
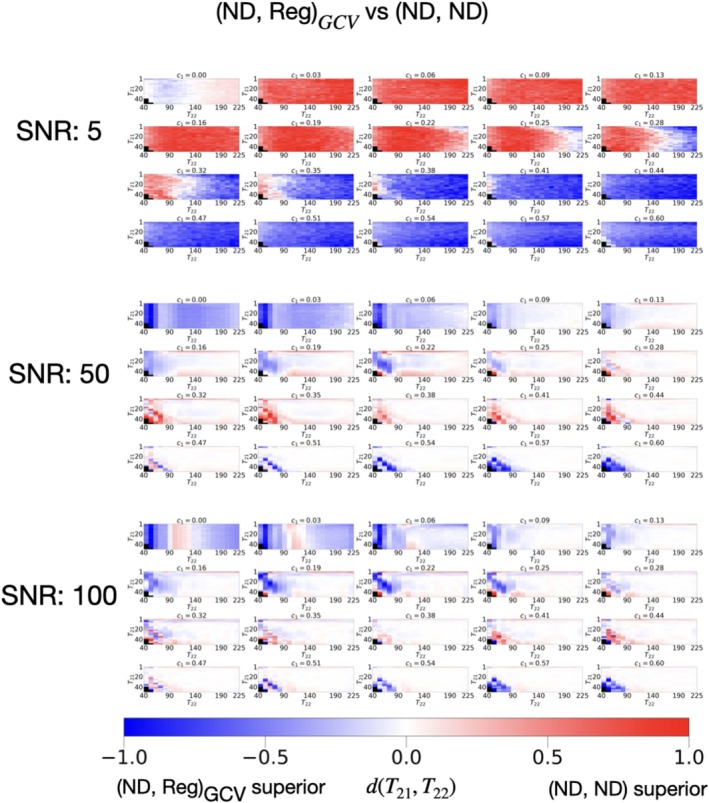
Top to bottom: Computing the differences in relative RMSE between (ND, Reg)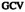 and (ND, ND). Each panel is an image over the $\left(T_{2,1},T_{2,2}\right)$ space where the color blue (resp. red) represents the superiority of (ND, Reg)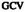 (resp. (ND, ND)) in estimating the MWF value given by $c_{1}$ denoted above the panel.

**FIGURE D2:**
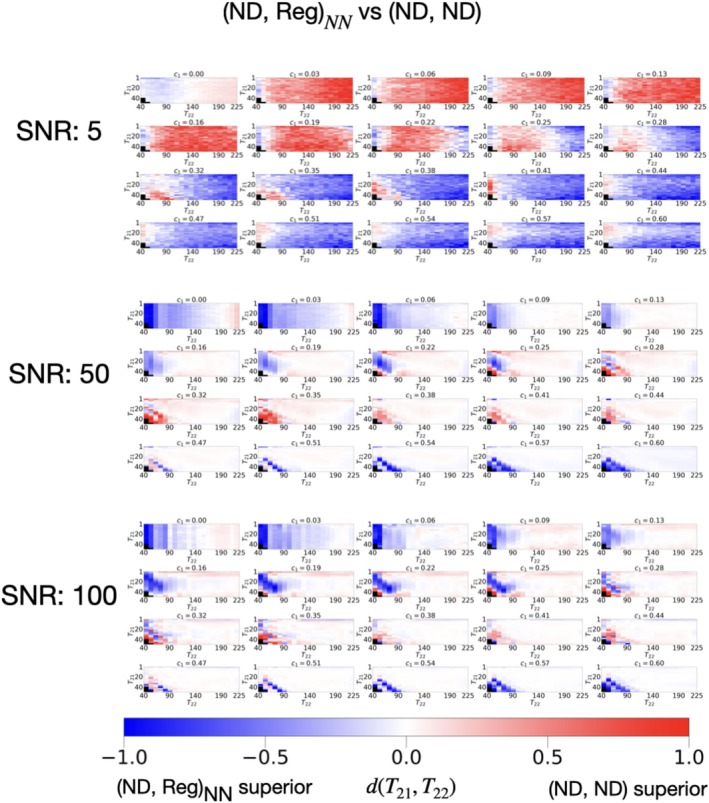
Results as for Figure A2 but for (ND, Reg)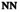.

**FIGURE E1:**
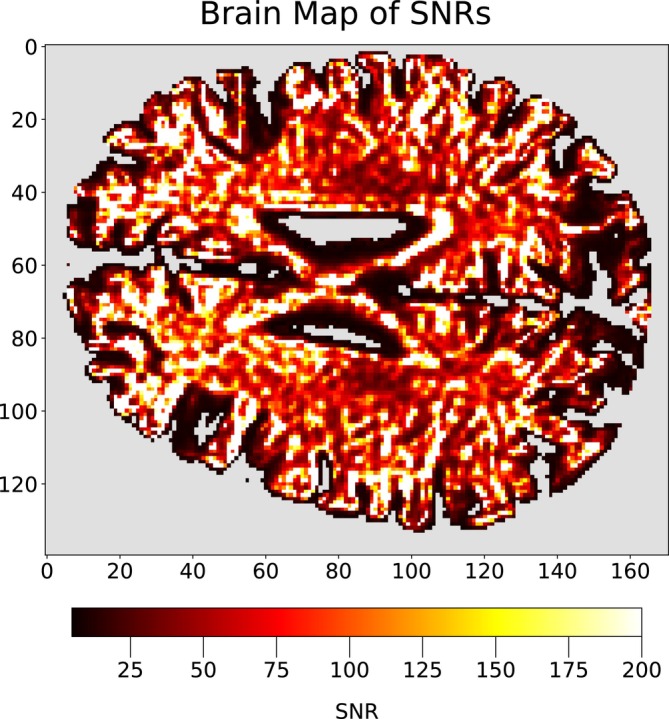
The SNR for each voxel was calculated by dividing the signal amplitude at the first echo time by the signal floor (offset value) as estimated with NLLS: $\frac{SA_{TE_{0}}}{offset/\sqrt{\pi /2}}$. Excluded voxels are colored gray.

## Data Availability

The data that support the findings of this study are openly available in a GitHub repository at https://github.com/ShashankSule/ILR‐for‐MWF.

## Appendix A Dataset Generation

We generate the training data used for $\lambda_{\text{NN}},\;{\text{(ND, Reg)}}_{\text{NN}}$, and (ND, Reg) as follows. For each parameter vector p in a uniformly spaced grid of possible parameter values specified in Table A1, we generate a noiseless signal G(p) using (3). Every noiseless signal is then corrupted with 1000 independent and identically distributed (iid) copies of Rician noise to generate noisy signals resembling experimental MRI data. The noisy signals are normalized to have the initial signal amplitude be 1.0 at the minimum echo time $TE_{0}=8.0$. Finally, we assemble all the noisy signals over all the parameter vectors into the set of training inputs denoted by $\left\{{\text{s}}_{i}\right\}$. Then, the parameters used for generating ${\text{s}}_{i}$ will be indexed accordingly and assembled into the set of training outputs given by $\left\{{\text{p}}_{i}\right\}$. To obtain $\lambda_{oracle}({\text{s}}_{i})$, we perform a grid search over $\lambda \in [10^{-7},10^{3}]$ for finding minimizers to (9). For obtaining $\lambda_{\text{GCV}}$, we employ another grid search in the range $\lambda \in [10^{-7},10^{3}]$ to find the minimizers in (28). Our procedure is summarized in Algorithm A1.

**TABLE A1:** Parameters defined for dataset creation.

Parameter	Symbol	Value
Number of signal acquisition times	${\text{N}}_{t}$	32
Lower and upper bounds on regularization parameter search space	$\log_{10}(\lambda_{oracle})$	−7, 3
Initial guess for $\lambda_{oracle}$‐solver (regularized nonlinear least squares solver)	**z**	$\left(T_{2,1}^{true},T_{2,2}^{true},c_{1}^{true}\right)$
Lower and upper bounds on target fraction of more rapidly decaying component	$c_{1}^{l},c_{1}^{h}$	[0.0, 0.6] (Training), [0.0, 0.5] (Validation and testing)
Fraction of more slowly decaying component	$c_{2}$	$1.0-c_{1}$
Lower and upper bounds on target $T_{2,1}$	$T_{2,1}^{l},T_{2,1}^{h}$	[1.0, 50.0] (Training), [5.0, 45.0] (Validation and testing)
Lower and upper bounds on target $T_{2,2}$	$T_{2,2}^{l},T_{2,2}^{h}$	(Training), [45.0, 200.0] (Validation and testing)
Number of uniformly spaced $T_{2}$ and $c_{1}$ values	${\text{N}}_{p}$	20 (per parameter)
Range of signal acquisition times		[8.0,256.0]
Number of noise realizations	$N_{\omega }$	1000 (training), 200 (validation), and 400 (testing)

Algorithm A1 Dataset generation.

1: Set dataPurpose ▹dataPurpose is training, validation, or testing
2: Initialize parameters from Table A1.
3:  dataPurpose $T:=\{(c_{1},T_{2,1},T_{2,2})\in [c_{1}^{l},c_{1}^{h}]\times [T_{2,1}^{l},T_{2,1}^{h}]\times [T_{2,2}^{l},T_{2,2}^{h}]:T_{2,1}\leq T_{2,2}\}$
4:  SNR
5: Initialize empty list for samples: samples = List$\left(N_{T}\cdot N_{\omega }\right)$
6: Seed random number generator: seed(dataPurpose)
7: **for** $[c_{1},T_{2,1},T_{2,2}]\in T$ **do**
8:  Compute noiseless signal: $\text{G}(c_{1},T_{2,1},T_{2,2}):={\left(c_{1}{\text{e}}^{\frac{-t_{n}}{T_{2,1}}}+(1.0-c_{1}){\text{e}}^{\frac{-t_{n}}{T_{2,2}}}\right)}_{n=1}^{N}$
9:  **for** noise realization $k=1,\text{…},N_{\omega }$, **do**
10:  Get noise realization $\eta ,\xi ∼𝒩\left(\text{0},\frac{1}{SNR^{2}}I_{N}\right)$
11:  Add Rician noise to noiseless signal:
  (A1)
  $$\text{s}=\sqrt{{\left(G(\text{p})+\xi \right)}^{2}+\eta^{2}}$$
12:  **for** regularization parameter $\lambda \in [10^{-7},10^{3}]$
13:  Solve Tikhonov‐regularized NLLS problem with curve_fit:
14:  ${\text{p}}_{\lambda }^{*}\equiv {\text{argmin}}_{\text{p}\in {\mathbb{R}}_{+}^{2}}({\left\|\text{G(p)}-\text{s}\right\|}_{2}^{2}+\lambda^{2}\|\text{p}{\|}_{2}^{2})$.
15:  **end for**
16:  $\lambda_{oracle}:={\text{argmin}}_{\lambda }({\left({\text{p}}_{\lambda }^{*}-{\text{p}}_{opt}\right)}^{2})$
17:
18:  Append $\left(G,\lambda_{oracle}\right)$ to samples
19:  **end for**
20: **end for**

## Appendix B NN Training

### B.1

In this paper, we trained four distinct NNs:

1. $\lambda_{\text{NN}}$ for estimating $\lambda_{oracle}$ from the noisy signal s,
2. (ND, Reg) for estimating ${\text{p}}_{true}$ from $\left(\text{s},G({\text{p}}_{\lambda_{\text{NN}}(\text{s})}(\text{s})\right)$,
3. (ND, Reg) for estimating ${\text{p}}_{true}$ from $\left(\text{s},G({\text{p}}_{\lambda_{\text{GCV}}(\text{s})}(]\text{s})\right)$, and
4. (ND, ND) for estimating ${\text{p}}_{true}$ from (s,s).

#### B.1 Training $\lambda_{\text{NN}}$

Here, we outline the procedure to train our $\lambda_{\text{NN}}$ to learn the optimal regularization parameter. The architecture of $\lambda_{\text{NN}}$ is provided in Figure B1 because it uses a combination of convolutional and fully connected layers. We train it for 30 epochs with the default parameters of an Adam optimizer, with a batch size 64, and a learning rate $10^{-5}$.

#### B.2 Training ILR Networks and (ND, ND)

Here, we outline the procedure to train our (ND, ND), (ND, Reg), and ${\text{(ND, Reg)}}_{\text{GCV}}$ models to learn the parameter estimate from the given input. We use a four‐layer fully connected NN with ReLU activations and dimensions 32→256→64→256→3. We train this network for 30 epochs, and a batch size of 64 with learning rate $10^{-5}$ using the default parameters of the Adam optimizer.

## Appendix C Estimating Time Decay Constants

### C.1

Although the MWF parameter $c_{1}$ was of central interest in this paper, the estimation of the time decay constants $T_{2,1}$ and $T_{2,2}$ is also an independently important problem in magnetic resonance. In Tables C1 and C2, we include results for estimating $T_{2,1}$ and $T_{2,2}$, respectively, noting that our ILR networks (ND, Reg) and (ND, Reg) output all three parameters at once. Therefore, no additional training or design is required to infer $T_{2,1}$ and $T_{2,2}$.

**TABLE C1:** Root mean squared error (RMSE) in $T_{2,1}$ for each parameter estimation method.

${\text{RMSE}}_{T_{2,1}}$/SNR	5.0	50.0	100.0
(ND, Reg)	14.45^a^	10.41^a^	8.55^a^
(ND, Reg)	14.45^a^	10.42	8.63
(ND, ND)	14.46	11.03	8.99
TR‐NLLS, $\lambda_{oracle}$	23.03	18.46	18.40
TR‐NLLS, $\lambda_{\text{GCV}}$	92.39	66.40	54.79
NLLS	$38.18\times 10^{6}$	$41.32\times 10^{5}$	$16.71\times 10^{5}$

^a^ Superior performance for the indicated SNR.

**TABLE C2:** Root mean squared error (RMSE) in $T_{2,2}$ for each parameter estimation method.

${\text{RMSE}}_{T_{2,2}}$/SNR	5.0	50.0	100.0
(ND, Reg)	36.77^a^	8.49^a^	5.31^a^
(ND, Reg)	36.80	8.50	5.31^a^
(ND, ND)	36.86	8.57	5.35
TR‐NLLS, $\lambda_{oracle}$	32.85	7.99	7.53
TR‐NLLS, $\lambda_{\text{GCV}}$	$15.29\times 10^{3}$	$59.20\times 10^{2}$	$53.68\times 10^{2}$
NLLS	$41.61\times 10^{7}$	$12.86\times 10^{7}$	$13.69\times 10^{7}$

^a^ Superior performance for the indicated SNR.

## Appendix D Disaggregating Table 2

### D.1

Although Table 3 shows that our method outperforms both plain deep learning and classical methods overall, it is of interest to define the regions of parameter space $\left(c_{1},T_{2,1},T_{2,2}\right)$ in which ILR is particularly beneficial over the plain neural network (ND, ND). To investigate this issue, we compared the performance of the two NNs, ${\text{(ND, Reg)}}_{\text{NN}}$ and ${\text{(ND, Reg)}}_{\text{GCV}}$ against (ND, ND) as follows. First, let $c_{1}^{\left(ND,Reg\right)},\;c_{1}^{\left(ND,ND\right)}$ be the $c_{1}$ parameter estimates produced by the (ND, Reg) and (ND, ND) networks, respectively, where the subscript NN or GCV has been omitted for clarity here and below. Now, given a fixed $c_{1}^{true}$, for each underlying pair $\left(T_{2,1},T_{2,2}\right)$, we calculate the differences between the relative RMSE of the two estimates:

(D1)
$$\hat{d}(T_{21},T_{22})=RMSE(c_{1}^{\left(ND,Reg\right)},c_{1}^{true})/c_{1}^{true}-RMSE(c_{1}^{\left(ND,ND\right)},c_{1}^{true})/c_{1}^{true},$$

(D2)
$$d(T_{21},T_{22})=\frac{\hat{d}(T_{21},T_{22})}{\underset{T_{21},T_{22}}{\max}|\hat{d}(T_{21},T_{22})|}.$$

Here, the RMSE is taken over 400 noise realizations present in the test set. From this, $\hat{d}(T_{2,1},T_{2,2})<0$ corresponds to (ND, Reg) outperforming (ND, ND) for the corresponding pair of $\left(T_{2,1},T_{2,2}\right)$. We also consider a normalized version of this error metric given by $d(T_{2,1},T_{2,2})$ described in (D2) where the values of $\hat{d}$ are rescaled to be in the interval [−1,1]. We visualize $d(T_{2,1},T_{2,2})$ in Figures D1 and D2. Our findings are as follows.

#### D.1 Low SNR

In the low SNR (SNR = 5.0) regime (first panels from top in Figures D1 and D2), the two (ND, Reg) networks markedly exceed the (ND, ND) networks in performance for the $c_{1}\geq 0.32$ region while the (ND, ND) networks attain superiority when $c_{1}\leq 0.32$. In this latter range of $c_{1}$ values, (ND, Reg) and (ND, Reg) perform slightly differently. Specifically, when $0.25\leq c_{1}\leq 0.32,\;{\text{(ND, Reg)}}_{\text{NN}}$ outperforms (ND, ND) for more values of $\left(T_{2,1},T_{2,2}\right)$ than (ND, Reg).

Thus, in regions of parameter space where (ND, Reg) outperforms (ND, ND), the ILR network with GCV selection given by (ND, Reg) attains greater accuracy than (ND, Reg) (thus registering a lower aggregate RMSE in Row 1 of Table 3). However, (ND, Reg) outperforms (ND, ND) for larger regions of parameter space than (ND, Reg).

#### D.2 Medium and high SNR

When SNR=50,100, both of the (ND, Reg) networks exhibit distinct patterns in three different ranges of $c_{1}$ space:

1. $0.0\leq c_{1}\leq 0.25$: In the low $c_{1}$ range the (ND, Reg) networks outperform (ND, ND). This gain in performance is specifically more pronounced in the $T_{2,2}-T_{2,1}\approx 20$ region, as evidenced by the diagonal blue bands in the lower left corners in the central cells of the SNR 50 and 100 panels in Figures D1 and D2. However, as the difference $T_{2,2}-T_{2,1}$ decreases further to zero, there is a greater prevalence of red pixels in the left corner of each cell, indicating that when the signal is nearly monoexponential, ILR likely introduces inaccuracies stemming from model misspecification. More generally, the difference between the decay times, $T_{2,2}-T_{2,1}$, is an indicator of the ill‐posedness of the biexponential problem. Our results in Figures D1 and D2 for this range of $c_{1}$ space may thus be qualitatively described as follows: In the regions of parameter space with a large weighting of the faster decay (i.e., $c_{1}\leq 0.25$), ILR is particularly advantageous when the problem features an intermediate level of ill‐posedness (i.e., $T_{2,2}-T_{2,1}\approx 20$). However, when the time constants are nearly identical, (ND, ND) exhibits superior performance.
2. $0.25\leq c_{1}\leq 0.4$: In the intermediate $c_{1}$ range, the (ND, ND) networks largely outperform the (ND, Reg) networks. Moreover, the (ND, ND) networks specifically attain lower RMSE in the ill‐posed region where $T_{2,1}\approx T_{2,2}$.
3. $0.4\leq c_{1}\leq 0.6$: This is the region of parameter space where the two time decays are nearly equally weighted. In this region of equal weighting, ILR is substantially better than plain neural network estimation, with the most significant improvements occurring in the ill‐posed regions. This phenomenon persists for both medium and high SNRs for both (ND, Reg) networks.

#### D.3 Does ILR Behave Like Classical Regularization?

For the NLLS estimator, regularization improves parameter estimation in the regions of ill‐posedness, that is, when $T_{2,1}$ and $T_{2,2}$ are within, roughly, a factor of 2 or 3 of each other. A natural question then is whether ILR acts as a regularizer for the parameter estimation problem; this can be evaluated by examining its performance in ill‐posed and well‐posed regimes of parameter space. In Figures D1 and D2, the lower left corners are the most ill‐posed regimes. As seen, the effect of ILR is to improve overall performance across the diagrams but without the improvement generally being concentrated in the ill‐posed regime.

## Appendix E Brain Data

### E.1 MRI Acquisition Protocol

After obtaining written informed consent, imaging was performed under our IRB protocol #08AG0238 for data collection, imaging was performed with a 3T Philips MRI system (Achieva, Best, The Netherlands), with an internal quadrature body coil for transmission and an eight‐channel phased array head coil for reception. We used the 3D GRASE (gradient and spin‐echo) sequence to acquire data from the brain of a healthy 41‐year‐old male. Echo data were acquired for 32 echoes, with acquisition times of $t_{n}=n\times \Delta t$ where the echo spacing Δt=11.3 ms. An echo planar imaging (EPI) acceleration factor of 3 was used, with a field of view (FOV) 278mm×200mm×30mm, over an acquisition matrix size of 185×133×10, for a voxel size of 1.5mm×1.5mm×1.5mm, with TR=1000ms. The image matrix was zero filled in k‐space to yield a 1mm×1mm×1mm reconstruction. The duration of the scan was approximately 10 min. Sampling was performed at echo peaks and formed the signal data for analysis.

### E.2 Noise Filtering and Quantification

Lacking a true “ground truth” for in vivo data, we used the NESMA filter [54] to obtain high SNR images from which to extract parameter values that serve as our (necessarily flawed) standard for comparison. This also provided a base image to which we added noise to achieve the levels of SNR as described in the main text, allowing us to implement a targeted application of an ILR network trained on data of low, medium, or high SNR. Figure E1 displays the calculated SNRs.

### E.3 Akaike Information Criterion (AIC)

We wish to apply regularization only to signals well‐described as biexponential rather than monoexponential. In the latter case, our biexponential model is undetermined, and the resulting parameter variances cannot be remediated by regularization or any other numerical approach. We used the AIC to identify biexponential signals. The AIC provides a criterion for determining whether the improved numerical fit that inevitably results from an increase in parameters for nested models is statistically meaningful. In the context of least squares regression, the AIC can be derived from the residual sum of squares (RSS). For a linear regression model with normally distributed errors, the likelihood L is proportional to $\exp\left(-\frac{RSS}{2\sigma^{2}}\right)$. This leads to the following expression for the AIC:

AIC=nlog(RSS/n)+2k

Here, n is the number of data points, RSS is the residual sum of squares, and k is the number of parameters. In the context of MWF imaging, we consider two competing models for explaining the data:

(E1)
$${\text{G}}_{monoexp}(t,c_{1},T_{2,1},b)=b+c_{1}\exp(-t/T_{2,1}),$$

(E2)
$${\text{G}}_{biexp}(t,c_{1},T_{2,1},c_{2},T_{2,2},b)=b+c_{1}\exp(-t/T_{2,1})+c_{2}\exp(-t/T_{2,2}).$$

Here, b is an offset, $c_{1},\;c_{2}$ are compartment sizes, and $T_{2,1}$ and $T_{2,2}$ are exponential time constants, as defined in the text. The AIC provides a method to compare the suitability of these models. In the context of MWF imaging each voxel can be regarded as containing a mono or biexponentially decaying signal corrupted by Rician noise. The RSS is not strictly an estimate of the lik`elihood of the data for non‐Gaussian noise, but our SNR range after NESMA filtering is sufficiently large that the difference between Gaussian and Rician noise can be neglected.

### E.4 Model‐Informed Machine Learning (MI‐ML) [30]

In MI‐ML, a neural network $f_{\theta }$ is trained with the following loss function given by a weighted sum of MSE loss with the Wasserstein‐1 distance:

(E3)
$$L_{MI-ML}(\theta ):=\frac{1}{n}\overset{n}{\underset{i=1}{\sum }}\|p_{true}^{i}-f_{\theta }(s^{i}){\|}_{2}^{2}+\alpha \frac{1}{n}\overset{n}{\underset{i=1}{\sum }}{𝒲}_{1}(p_{true}^{i},f_{\theta }(s^{i})).$$

To define ${𝒲}_{1}(p_{true}^{i},f_{\theta }(s^{i}))$ between the true parameters and the estimated parameters, we first summarize the **Wasserstein‐1 distance** (also called the earth mover's distance) between two probability distributions μ,ν∈𝒫(ℝ):

(E4)
$$W_{1}(\mu ,\nu )\;=\;\underset{\gamma \in \Pi (\mu ,\nu )}{\inf}\int_{\;\mathbb{R}\times \mathbb{R}}|x-y|\;d\gamma (x,y),$$

where Π(μ,ν) denotes the set of all couplings of μ and ν. If $F_{\mu }$ and $F_{\nu }$ denote the cumulative distribution functions (CDFs) of μ and ν, then

(E5)
$$W_{1}(\mu ,\nu )\;=\;\int_{\;-\infty }^{\infty }\left|F_{\mu }(x)-F_{\nu }(x)\right|\;dx,$$

that is, the Wasserstein‐1 distance is exactly the $L^{1}$‐distance between the two CDFs. Letting $p_{est}=(c_{1}^{est},T_{2,1}^{est},T_{2,2}^{est})=f_{\theta }(s^{i})$, we may construct the distribution ${𝒫}_{est}:=c_{1}^{est}\delta_{1/T_{2,1}^{est}}+(1-c_{1}^{est})\delta_{1/T_{2,2}^{est}}$ (and similarly construct ${𝒫}_{true}$ from $p_{true}$. Then,

(E6)
$${𝒲}_{1}(p_{true},p_{est}):={𝒲}_{1}({𝒫}_{true},{𝒫}_{est}).$$

Note that ${𝒲}_{1}({𝒫}_{true},{𝒫}_{est})$ scales like $O(c_{1}/T_{21})$ and is therefore at an order of magnitude of $10^{-2}$. To keep this order of magnitude commensurate with the MSE loss, we use α=50 so that all terms are O(1).
